# Mental Health, Loneliness and Urbanicity – A Cross-Sectional Network Analysis in a Nationally Representative Sample

**DOI:** 10.1192/j.eurpsy.2025.284

**Published:** 2025-08-26

**Authors:** D. Ochnik, M. Budziński, A. Szostak, Y. Vitkovskyi, M. Wierzbik-Strońska, E. Rojczyk, P. Nagel

**Affiliations:** 1 Medicine; 2 Social Sciences and Humanities; 3 Architecture, Civil Construction, and Applied Arts, Academy of Silesia, Katowice, Poland

## Abstract

**Introduction:**

With increasing urbanization, more people are exposed to mental health risk factors stemming from the urban, social or physical environment. However, research on the connection between urban living and mental health remains unclear.

**Objectives:**

This study aims to explore environmental and social risk factors for mental health issues using network analysis.

**Methods:**

The study was conducted among a nationally representative sample of 2,701 habitants of Poland (51% of women). The measurements used were PHQ-9, UCLA, Neighbourhood Cohesion (Neighbourhood Belonging and Social Cohesion), REAT 2.0 (Quality of architecture conditions in neighbourhood area), distance and frequency use of blue, green, and urban public areas, Self-Rated Health, Physical Activity, urbanicity, size of place of residence per person and sociodemographic survey (age, education, income). We used a sparse Gaussian graphical model (GGM) with a graphical lasso with an EBICglasso estimator.

**Results:**

We showed that urbanicity and physical environment were linked to mental health issues via neighbourhood cohesion and loneliness in the estimated network. Depression and anxiety were the nodes with the highest centrality strength and expected influence. Blue and green areas usage also had high centrality strength. Urbanicity played an important role as a bridge between the network nodes and had a high strength score. Physical health with blue and green areas frequency use had the highest closeness centrality score.

**Conclusions:**

We revealed the connections among mental health, loneliness, social cohesion, and various environmental factors, particularly urbaicity. This will enhance our understanding of mental health risks and protective factors.

This study is a part of the “Urbanization and Health”( NdS-II/SN/0391/2024/012) project financed by state budget funds granted by the Minister of Education and Science within the framework of the Science for Society II Program in Poland.

**Disclosure of Interest:**

None Declared

